# Optimal treatment for obsessive compulsive disorder: a randomized controlled feasibility study of the clinical-effectiveness and cost-effectiveness of cognitive-behavioural therapy, selective serotonin reuptake inhibitors and their combination in the management of obsessive compulsive disorder

**DOI:** 10.1097/YIC.0000000000000237

**Published:** 2018-09-27

**Authors:** Naomi A. Fineberg, David S. Baldwin, Lynne M. Drummond, Solange Wyatt, Jasmine Hanson, Srinivas Gopi, Sukhwinder Kaur, Jemma Reid, Virender Marwah, Ricky A. Sachdev, Ilenia Pampaloni, Sonia Shahper, Yana Varlakova, Davis Mpavaenda, Christopher Manson, Cliodhna O’Leary, Karen Irvine, Deela Monji-Patel, Ayotunde Shodunke, Tony Dyer, Amy Dymond, Garry Barton, David Wellsted

**Affiliations:** a Hertfordshire Partnership University NHS Foundation Trust, Rosanne House, Parkway, Welwyn Garden City; b University of Hertfordshire, Hatfield; c University of Cambridge Clinical Medical School, Addenbrookes Hospital, Cambridge; d Clinical and Experimental Sciences, Faculty of Medicine, University of Southampton; e Southern Health NHS Foundation Trust, College Keep, Southampton; f London and St George’s Mental Health NHS Trust; g St George’s Hospital, University of London, London; h Norwich Medical School, Faculty of Medicine and Health Sciences, University of East Anglia, Norwich, UK

**Keywords:** cognitive behaviour therapy, feasibility, health economic, obsessive–compulsive disorder, randomized, sertraline

## Abstract

Established treatments for obsessive compulsive disorder (OCD) include cognitive behaviour therapy (CBT) and selective serotonin reuptake inhibitor (SSRI) medication. Combined treatment may outperform monotherapy, but few studies have investigated this. A total of 49 community-based adults with OCD were randomly assigned to CBT, SSRI, or SSRI+CBT. Sertraline (50–200 mg/day) was given as the SSRI for 52 weeks. A 16-h-manualized individual CBT was delivered over 8 weeks with four follow-up sessions. Assessors were ‘blinded’ to treatment allocation. A preliminary health economic evaluation was conducted. At week 16, combined treatment (*n*=13) was associated with the largest improvement, sertraline (*n*=7) the next largest and CBT (*n*=9) the smallest on the observed case analysis. The effect size (Cohen’s *d*) comparing the improvement in Yale Brown Obsessive Compulsive Scale on CBT versus combined treatment was −0.39 and versus sertraline was −0.27. Between 16 and 52 weeks, the greatest clinical improvement was seen with sertraline, but participant discontinuation prevented reliable analysis. Compared with sertraline, the mean costs were higher for CBT and for combined treatment. The mean Quality Adjusted Life Year scores for sertraline were 0.1823 (95% confidence interval: 0.0447–0.3199) greater than for CBT and 0.1135 (95% confidence interval: ‑0.0290–0.2560), greater than for combined treatment. Combined treatment appeared the most clinically effective option, especially over CBT, but the advantages over SSRI monotherapy were not sustained beyond 16 weeks. SSRI monotherapy was the most cost-effective. A definitive study can and should be conducted.

## Background

Obsessive compulsive disorder (OCD) is a common, disabling, relapsing psychiatric illness (Skoog and Skoog, 1999). Combining pharmacotherapy with cognitive behaviour therapy (CBT) has been considered superior to either treatment given alone, but is probably more costly, and few controlled studies have addressed this question (Fineberg *et al.*, 2013). Moreover, interpretation of the many published studies is compromised by poor study design (Skapinakis *et al.*, 2016a, 2016c). UK NICE guidelines (National Institute for Health and Care Excellence, 2006) recommend monotherapy with either CBT [including exposure and response prevention (ERP)] or selective serotonin reuptake inhibitor (SSRI) medication as standard first-line treatments, with combined therapy (CBT+SSRI) reserved for patients with more severe or enduring illness. This staging is, however, largely based on clinical consensus (level IV evidence). There is additional uncertainty relating to the quality of life (QOL) gain and cost-effectiveness associated with the different treatment options (National Institute for Health and Care Excellence, 2006; Skapinakis *et al.*, 2016b).

Robust data from randomized controlled studies (RCTs) in OCD suggest that continuing an SSRI protects against relapse (Fineberg *et al.*, 2007), whereas discontinuation contributes to relapse and reduction in life quality (Hollander *et al.*, 2010). Continuation-treatment with SSRI is, therefore, a recognized strategy for long-term well-being (Fineberg *et al.*, 2015). In contrast, little is known from RCTs about long-term outcomes of OCD following the termination of a course of CBT. There remains a need for studies to determine the effectiveness of CBT ‘booster-regimes’ as an alternative means to prevent relapse (Fineberg *et al.*, 2013). Taking this uncertainty into consideration, there is a pressing need for RCTs of combined treatment versus monotherapy of adequate duration to meaningfully guide optimal service delivery for people with OCD.

## Aims and objectives

This 52-week feasibility study was designed to inform the design of a definitive RCT, to determine the clinical-effectiveness and cost-effectiveness of combining CBT with SSRI versus either treatment when given alone, in patients not known to be treatment resistant. We, therefore, included a broad range of outcomes covering several modalities including the number of eligible patients randomized, clinical outcomes, premature discontinuation rates, tolerability across treatment arms, resource use and QOL.

A key objective was to estimate the relative effect size of each arm, for which we used an observed case analysis of the variation in the primary endpoint – defined as the change in total Yale Brown Obsessive Compulsive Scale (Y-BOCS; Goodman *et al.*, 1989) score, both within and between the three treatment arms at week 16 and beyond, in order to estimate the sample size required to power a definitive trial.

### Methods and analysis

The trial was approved by the East of England NHS ethics committee, REC reference 13/EE/0431. Written informed consent was obtained from all participants, both for screening and for the treatment phase. The trial strictly followed Good Clinical Practice regulations.

### Design

This was a three-arm, multicentre, randomized, feasibility trial. The treatment arms were SSRI monotherapy, CBT monotherapy, and the combination of SSRI and CBT. The trial assessed adult participants (18–65 years) over 52 weeks with measures taken at weeks 0, 2, 4, 8, 16, 32 and 52. To minimize bias, the outcome assessments were performed by researchers who were separate from the clinical teams and blinded to treatment allocation.

### Location

The study took place at three UK centres accessing large populations of OCD patients: Hertfordshire Partnership University NHS Foundation Trust; South West London and St George’s NHS Mental Health Trust and Southern Health NHS Foundation Trust. Depending on local service configuration, aspects of study treatment for patients with moderate-intensity OCD were delivered in the primary care (immediate access to psychological therapies) setting to model usual practice.

### Participant selection

Participants were male or female treatment-seeking community-based patients aged 18–65 years with an OCD of at least moderate severity and a documented duration of symptoms greater than one year taken from their medical records. They were identified from routine trust referrals, active recruitment from usual referral sources including primary healthcare services [e.g. general practitioners (GPs), immediate access to psychological therapies services], community mental health clinics, psychotherapy waiting lists and advertisement through national OCD and other charities, as well as through local media adverts including websites, newspapers and radio programmes.

### Inclusion

Participants needed to have a *Diagnostic and Statistical Manual of Mental Disorders*, 4th ed. (DSM-IV) diagnosis of OCD (American Psychiatric Association, 1994), determined by a doctor using the Mini International Neuropsychiatric Inventory for DSM-IV (Sheehan *et al.*, 1998) and a baseline total score of more than 16 on the Y-BOCS. Participants with comorbid psychiatric disorders were allowed to enter the study, subject to the exclusion criteria listed below, and provided OCD was judged to be the primary focus of clinical intervention.

### Exclusion

Those with a history of psychotic disorder, Tourette syndrome (tic disorders not amounting to Tourette syndrome were allowed), organic mental disorder, psychosurgery, personality disorder of borderline or histrionic type, or alcohol/substance-abuse disorders within the past 12 months were not recruited. In addition, those with severe depressive symptoms, defined by a Montgomery–Asberg Depression Rating Scale (MADRS; Montgomery and Åsberg, 1979) score of more than 30 at baseline, or those actively planning suicide (scoring >4 on item 10 of MADRS), or judged by the clinician to be at significant risk of self-harm, were excluded.

We also excluded individuals with treatment-resistant OCD, defined as failing to respond to more than one previous adequate (>12 weeks) trial of CBT involving ERP from an accredited (British Association of Behavioural and Cognitive Psychotherapies approved or equivalent) therapist, or failing to respond to more than 2 adequate (>12 weeks) trials of any SSRI or clomipramine taken at optimal doses (if <maximum SPC dose, evidence of intolerance of the higher dose was needed) with adequate adherence.

Those needing regular psychotropic drugs other than study medication during the trial (except for hypnotics, which were allowed, provided the dose had been stable for at least 12 weeks, and remained so throughout the study period), or needing regular specified medication that might interact adversely with sertraline were also excluded, as were those with acute or unstable physical illness, women of child-bearing age who were not using reliable contraceptive methods, those who for individual reasons would find it difficult to comply with the treatment programme, including the washout period, and those who were judged to have insufficient understanding of English to participate in treatment or provide informed consent.

### Screening

Interested individuals were given a brief explanation of the trial, and a preliminary assessment of eligibility was undertaken. Potentially eligible and willing participants were provided with a patient information sheet and an appointment for a screening visit, being allowed at least 24 h for further consideration. At the screening visit, the patient was fully assessed by members of the research team, including a psychiatrically qualified research doctor who confirmed eligibility, on the basis of the inclusion and exclusion criteria, and obtained written consent. Depending upon the medical history, the need for a ‘washout period’ was ascertained. Those not needing washout were randomized, and baseline assessments were made during this visit. For those requiring a ‘wash-out’, the previous medication was discontinued according to standard guidance: individuals were then reassessed after a drug-free period, normally lasting one week (in the case of fluoxetine, 6 weeks).

Clinical contact was maintained throughout.

### Randomization

An independent online randomization service was provided by the Norwich Clinical Trials Unit (University of East Anglia). Participants were randomized to one of the treatment arms in a ratio of 1 : 1 : 1, and the patient was informed of the allocated treatment. Patients were specifically asked not to discuss their treatment allocation with the ‘blinded’ research assistant who conducted the subsequent ratings. They were provided with 24-h clinical contact information.

### Interventions

Participants were randomized to one of three interventions, designed to approximate to normal UK-prescribing and CBT practice:

1. Sertraline (50–200 mg) (group 1): 30-min outpatient visits with the doctor took place at weeks 0, 2, 4, 8, 16, 24, 32 and 52, at which sertraline was prescribed, and the effects, adverse events (AEs) and dosage were reviewed. From week 0, sertraline was flexibly titrated upwards from 50 to 200 mg, in accordance with the licence, and guided by tolerability and clinician-patient judgement. Doses could be adjusted upwards or downwards for the first 8 weeks, aiming for the highest tolerated dose, after which the dosage of medication was fixed until week 52. No CBT was provided, and doctors were trained to avoid covert discussion of CBT exercises.
2. CBT with ERP (group 2): CBT incorporated cognitive and behavioural interventions, including graded ERP, with individual face-to-face contact and homework assignments, as recommended by National Institute for Health and Care Excellence (2006). Treatment was manualized and adapted from Steketee (1993). In accordance with NICE guidance and with the level of illness severity of the OCD patients (Y-BOCS>16; not treatment resistant), a moderate-intensity CBT package was offered, comprising eight sessions of individual treatment, each lasting 2 h, to be delivered over 8 weeks. Four 1-h follow-up sessions were provided at weeks 16, 24, 32 and 52.
3. All CBT therapists were accredited by the British Association of Behavioural and Cognitive Psychotherapies or an equivalent professional body. Therapists received refresher training in CBT with ERP and training in the manual from one of the principal investigators with special expertise in CBT and ERP (L.D.). Therapists underwent peer group supervision on a regular basis. In addition, a sample of CBT sessions were audiotaped for fidelity, which was assessed for quality by L.D.
4. Sertraline (50–200 mg) plus CBT with ERP (group 3): Participants received both sertraline and CBT as per groups one and two.

At each study visit, patients were encouraged to return any unused medication, and a pill count was performed. For patients receiving CBT, information on attendance at scheduled therapy sessions, length of sessions and completion of homework was recorded.

At the end of study treatment, patients were referred to differing services, depending on their clinical status. In the case of premature discontinuation, patients were invited to continue with study assessments by the research assistant for research assessments until week 52, for research purposes only.

### Outcomes and endpoints

Randomized participants were evaluated at weeks 0, 2, 4, 8, 16, 32 and 52 by research assistants who were blinded to the treatment allocation. Every attempt was made to preserve rater blindness. The following outcomes were evaluated:

1. Primary outcomes: variation of the Y-BOCS as the primary outcome measure, both within and between the treatment arms, at week 16 (primary endpoint) and at week 52 (final endpoint).
2. Secondary outcomes: variation of the following outcomes on patient selection and on the primary endpoint, to inform minimization strategies in the subsequent definitive trial, and the need for adjusted and stratified analysis.
  - CGI Severity Scale and CGI Improvement Scale (Guy, 1976).
  - Sheehan Disability Scale (SDS) (Sheehan *et al.*, 1996).
  - MADRS (Montgomery and Åsberg, 1979).
  - Autism quotient (Baron-Cohen *et al.*, 2001) and performance on a computerized neurocognitive battery– to be reported separately.
3. At weeks 0, 16 and 52, the self-report EuroQoL EQ-5D-3L (Brooks, 1996) was administered. Treatment costs were assessed from an NHS and personal social services (PSS) perspective using an adapted version of the Client Service Receipt Inventory (Beecham and Knapp, 1992).
4. Treatment-related adverse effects were measured via direct interview and grouped according to MeDRA terminology (Brown *et al.*, 1999). Tolerability measures including dropout rates and reasons were recorded.

Every effort was made to assess patients on the exact scheduled day; however, assessments could be made ±3 days outside this.

### Sample size calculation

Cocks and Torgerson (2013) recommend estimating the required sample size for a feasibility study based on a one-sided 80% confidence interval (CI) designed to exclude the minimum difference of clinical interest in the primary outcome measure. For the Y-BOCS, we estimated a typical pooled SD to be 8, giving an effect size of 0.25 for a two-point change and an effect size of 0.375 for a three-point change, equating to a required sample size of 251/arm. Cocks and Torgerson (2013) also recommend the sample size for a pilot study to amount to 9% of the estimated sample size. Thus, the range of required sample size was between 10 and 22 patients/arm, given the range of the expected effect. The sample size for this study was, therefore, set at 20/arm, assuming a total of 60 participants randomized and 45 patients completing the trial at least to 16 weeks, which we defined as the primary endpoint, to provide a robust estimate excluding a minimum change of three points in the worst case.

### Analysis

OTO was designed as a feasibility study, with the aim of ascertaining the relative effect sizes associated with the different treatment arms using observed cases, representing the most sensitive analysis, as it involves the fewest statistical assumptions. We also conducted sensitivity analyses of the ‘intent to treat’ (ITT) population to examine the extent to which the observed case findings could be confirmed, although the small sample size compromized the robustness of these additional analyses. The ITT analysis was evaluated as a mixed model with unstructured variance and appropriate interaction terms.

### Economic evaluation

The health economics component sought to measure the level of participant resource use and QOL within each of the three treatment options (sertraline, CBT and sertraline plus CBT).

All costs were estimated for the 2015/2016 financial year, from an NHS and PSS cost perspective. In terms of intervention costs, sertraline appointments were made with a specialist psychiatric registrar, who recorded attendance and sertraline dosage prescribed. Appointments were assumed to last 30 min, with an additional 30 min of noncontact time and 5 min of supervision (with a consultant psychiatrist), per appointment. CBT therapists recorded attendance and the contact time of each session. In addition, we assumed that there was 1 h extra of noncontact staff time per session, and that two CBT therapists per site received 2 h of joint CBT training, and 1 h supervision per month, from a consultant psychiatrist. However, research costs, for example, the time associated with the completion of outcome measures, were not included, as these would not be incurred if this intervention was provided in the NHS.

At baseline and subsequent in-person follow-up visits (16 and 52 weeks), participants were asked whether they had received any of a selection of other healthcare-related services (over a common time period of the previous 16 weeks), and to list the associated level of resource use (if applicable).

Unit costs (Table 1) were assigned to all items of resource use, and these costs were then summed in order to estimate the total per participant cost, wherein the total cost of both CBT training and supervision was equally apportioned across all those allocated to a CBT option.

**Table 1:**
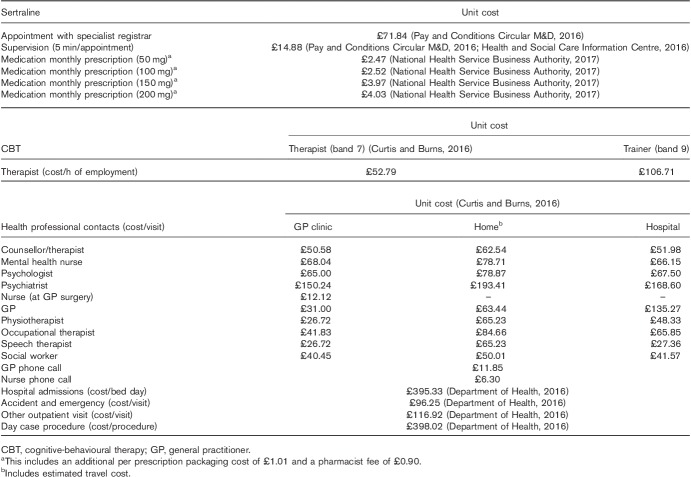
Estimated unit costs, with associated sources

QOL was measured using the EQ-5D-3L (Brooks, 1996) at baseline, 16 and 52 weeks. Responses were converted into utility scores (a scale wherein 0 is equal to death and 1 is full health) (Drummond *et al.*, 2015) using the York A1 tariff (Dolan, 1997), and Quality Adjusted Life Year (QALY) scores were subsequently calculated for the 52-week follow-up period, using the total area under the curve approach (Manca *et al.*, 2005).

Analysis included the estimation of completion rates and large cost drivers to inform the decision as to how costs and benefit should be collected as part of any future definitive study. A preliminary within-trial assessment of cost-effectiveness was also conducted (no discounting was undertaken, as time period was 1 year), on the basis of a complete-case approach (Briggs *et al.*, 2003), whereby participants were only included if the annual overall cost (the average cost of the first and last 16 weeks was used to estimate levels of resource use between weeks 16 and 36) and QALY score could be estimated. A bivariate regression (Willan *et al.*, 2004) was then undertaken to estimate the mean difference in overall cost and mean difference in QALYs between each of the three different treatment options. Cost and effect regression analyses were run simultaneously, with age, sex, ethnicity, marital status, living situation and education included as covariates, as well as the baseline EQ-5D score for the QALY regression. To estimate which option constituted ‘best value for money’, dominated options were ruled out on the basis that another option had both a higher mean effect and a lower mean cost. The incremental cost-effectiveness ratio (Drummond *et al.*, 2015) (mean incremental cost/mean incremental effect) was subsequently estimated for the remaining options, wherein an incremental cost-effectiveness ratio below £20 000 was considered to constitute value for money (Briggs *et al.*, 2002; National Institute for Health and Clinical Excellence, 2008). To estimate the associated level of uncertainty, the cost-effectiveness acceptability curve, which depicts the probability of an intervention being cost-effective at various ‘willingness to pay’ thresholds (Briggs *et al.*, 2002), was also estimated.

## Results

### Recruitment

**Fig. 1:**
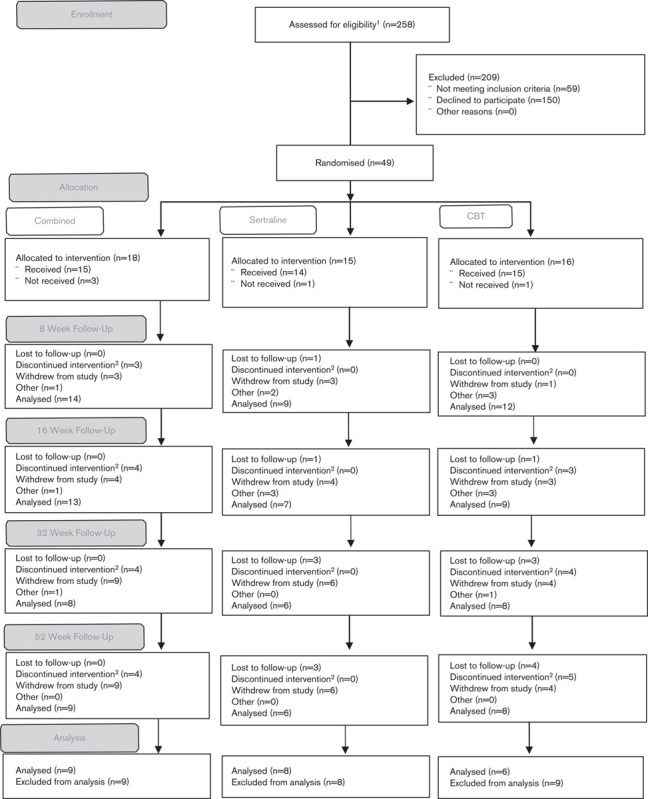
Study flow chart. ^1^Total *N* is defined as all unique patient identifiers on the study database. ^2^Patients withdrew from treatment, but agreed to be followed-up. ^3^Other is defined as patients who did not attend assessment at that time point.

A total of 258 patients were assessed for eligibility, of whom 59 were excluded and 150 declined to participate (Fig. 1). A total of 66 patients were screened, 10 were excluded and seven withdrew before randomization. A total of 49 patients were randomized (Hertfordshire Partnership University NHS Foundation Trust: 24; South West London and St George’s NHS Mental Health Trust: 8; Southern Health NHS Foundation Trust: 17), of whom 28 were female individuals. The major referral source for these patients was secondary care psychiatric services (*n*=21).

### Baseline characteristics

The three groups were reasonably well matched at baseline (Table 2). Overall, there was a slight preponderance of female individuals, and most patients were educated at least to university degree level. The mean total Y-BOCS score of 26.7 (SD: 7.5) indicated moderately severe OCD. Comorbid depression was diagnosed in 14 patients, although the mean total MADRS score of 16.1 (SD: 10.1) indicated that the severity of depression was not high in most cases. As expected, the most common psychiatric comorbidity was anxiety disorder (*n*=28). Also present were hoarding disorder (*n*=7), body dysmorphic disorder (*n*=5), obsessive–compulsive personality disorder (*n*=3), skin-picking disorder (*n*=2), hair-pulling disorder (*n*=2) and eating disorder (*n*=1). Patients were moderately impaired on the SDS, consistent with the community-based nature of the sample. However, the majority were single or divorced (39/49), consistent with the high celibacy rates seen in OCD.

**Table 2:**
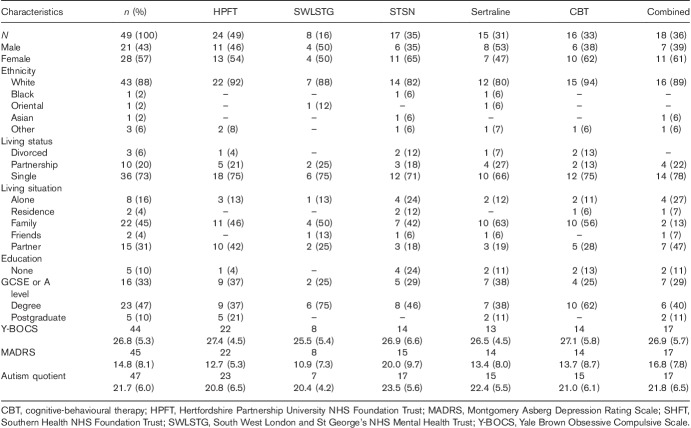
Baseline patient characteristics

### Acceptability

Five patients did not start the allocated study therapy after randomization: one CBT and one sertraline and three combined (one combined patient completed CBT only). Two patients cited not wanting the allocated treatment, and one not wanting to undergo washout, as their reasons for nonparticipation.

### Delay to starting treatment

There was on average an ~4-week delay in starting CBT, owing to logistical constraints; hence, most participants completed CBT between week 8 and week 16.

### Retention

Of the 44 participants starting treatment, 35 (79.6%) completed 8 weeks on the study and 29 (65.9%) reached the week 16 endpoint (CBT=9, SSRI=7, CBT+SSRI=13). Only two of the 15 participants who received their allocated treatment and discontinued the study within the first 16 weeks were receiving CBT+SSRI, compared with six receiving CBT and seven receiving SSRI, suggesting a possible advantage in terms of retention for the combined treatment group during the acute treatment phase. However, the number of patients completing 16 weeks fell short of the study power set in the protocol, for which we required 15 patients to complete each study arm (or 45 in all).

Between 16 and 52 weeks, only a further six patients discontinued. At 52 weeks, 23 (52.3%) participants remained in the study (CBT=8, SSRI=6, CBT+SSRI=9).

### Reasons for discontinuation

Reasons for premature withdrawal were obtained in 19 of the 26 cases, of which seven appeared directly related to study procedures. Four participants found randomization to SSRI unacceptable, one found washout of SSRI unacceptable, and two found attendance at study assessments unacceptable.

### Adherence

The majority of patients on SSRI [29/33 (88%)] took study medication as prescribed, as measured by pill counts on returned packets. The mean daily dose of sertraline prescribed in the SSRI group at week 52 was 166.67 mg, and, in the combination group, it was 100.00 mg.

Adherence to CBT was also found to be acceptable, according to a predetermined criterion of 75% compliance, in 24 (71%) of the 34 cases. For those patients who did not withdraw prematurely, almost all study assessments were completed. Fidelity to CBT was confirmed by random sampling of audiotaped sessions and assessment by a CBT expert (L.D.).

Two patients randomized to combination therapy and one patient randomized to SSRI monotherapy took no study medication (100% noncompliant), as they did not want to take medication. One patient randomized to combination therapy stopped study medication but continued to take a lower dose of sertraline prescribed by their GP. Four patients in the CBT arm started medication [three sertraline (2 at week 8, 1 at week 16), 1 fluoxetine at week 24] while still on study treatment.

### Clinical outcomes

#### Primary outcome (Y-BOCS)

All treatment arms were associated with a numerical improvement in total Y-BOCS scores over the course of the 52-week study (Table 3). At week 16 (primary endpoint), there was a substantial advantage for the combination treatment over CBT (Cohen’s *d*=0.39, 95% CI: −0.47 to 1.24) and a more modest advantage for SSRI over CBT (*d*=0.27, 95% CI: −0.73 to 1.3). At week 32 and at week 52, however, there was a marked advantage for SSRI monotherapy when compared with both CBT [*d* (week 32)=0.57, 95% CI: −0.52 to 1.7; *d* (week 52)=0.56, 95% CI: −0.53 to 1.6] and combination treatment [*d* (week 32)=−0.49, CI: −1.6 to 0.59; *d* (week 52)=−0.44, 95% CI: −1.5 to 0.61] (Table 3).

**Table 3:**
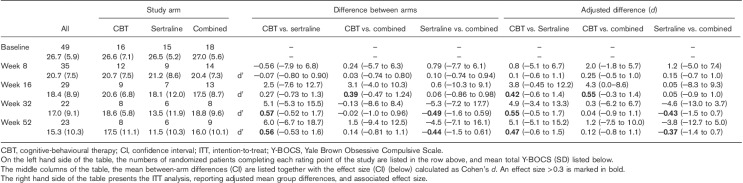
Total Y-BOCS scores on CBT, sertraline and combined treatment; observed case analysis and intent-to-treat analysis

#### Intent-to-treat analysis for the primary outcome

The findings of the ITT analysis are shown in Tables 3, 5 and 6, showing the difference between the arms as an adjusted difference. While the adjusted group differences do vary from the observed difference (in the middle section in each table), the overall pattern of the outcome is very similar to the observed group differences, and no change in the interpretation of the data is necessary. The largest change is seen for the MADRS in the comparison between CBT and the combined study arms. In this case, a small advantage for combined therapy over CBT in the first 16 weeks of the study is increased in the adjusted analysis, but falls to a negligible difference at 52 weeks.

### Secondary outcomes

#### Y-BOCS response rate

The number of full and partial responders was calculated, defined, respectively, as 35 and 25% improvement in the baseline Y-BOCS. The results showed a tendency for either full or no response, with an advantage for combination treatment at week 16, and an equivalent advantage for sertraline or combination treatment over CBT at week 52 (Table 4).

**Table 4:**
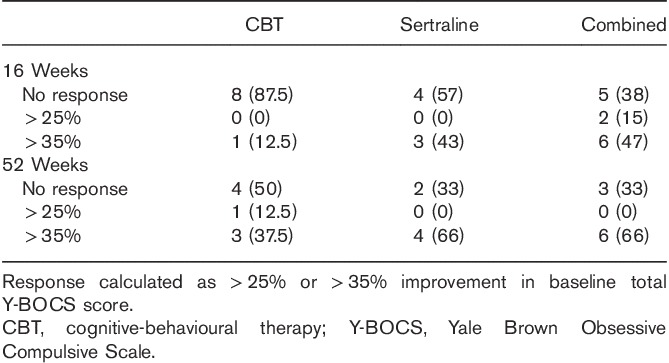
Responder rates

#### MADRS

Although starting from a numerically lower baseline, the MADRS scores for those in the CBT arm did not improve and were numerically higher than baseline at both week 16 and week 52. In contrast, MADRS scores in the sertraline arm improved substantially over the first 8 weeks of treatment and remained low (<10) between the 8-week and the 52-week endpoints. In the combination arm, the magnitude of the improvement in the MADRS was less than that for sertraline monotherapy, and scores worsened after 16 weeks (Table 5).

**Table 5:**
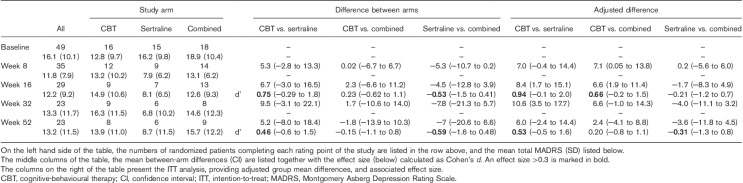
Total MADRS scores on CBT, sertraline or combined treatment

At 16 weeks, the effect size of the difference between the sertraline [mean MADRS=8.1 (SD: 6.5)] and CBT arms [mean MADRS=14.9 (SD: 10.6)] was 0.75 (95% CI: −0.29 to 1.8). At week 52, the effect size of the difference was 0.46 (95% CI: −0.62 to 1.5). Similarly, at week 16, the effect size of the difference between the sertraline and the combination arms [mean MADRS=12.6 (SD: 9.3)] was 0.53 (95% CI: −1.5 to 0.41), and, at week 52, it was 0.59 (95% CI: −1.6 to 0.48).

#### CGI Severity

Over the 16-week treatment phase, CGI scores improved similarly in the SSRI and combination arms, but not in the CBT arm. After week 16, however, the SSRI and the CBT arms showed further improvement, but the combination group did not. At week 52, the mean CGIs scores on sertraline, CBT and combined treatment were respectively 2.2 (SD: 1.2), 2.9 (SD: 1.8) and 3.2 (SD: 1.7) (Table 6).

**Table 6:**
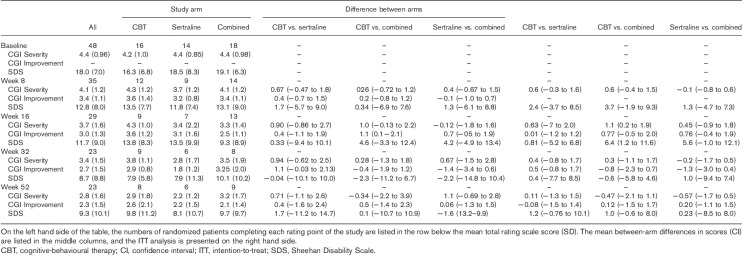
CGI Severity, CGI Improvement and Sheehan Disability Scale scores on CBT, sertraline or combined treatment

#### CGI Improvement

Compared with baseline, at week 16, all three arms were on average ‘minimally improved’ on the CGI Improvement, with a numerical advantage for the combination arm. By week 52, further improvement was seen to the extent that the SSRI and combination groups were ‘much improved’ to a similar degree, whereas the CBT group was ‘minimally improved’ (Table 6).

#### SDS

The SDS is a self-rated measure of impairment, scored in three domains: family, social and working life, which were totalled to provide a composite score. In the case of missing data in one of the three domains, a pro rata score for that domain was calculated. As some patients did not work, the work domain was not scored, and, in these cases, a pro rata score was estimated, wherein one of the remaining domains was completed (Table 6).

Patients in all three groups showed a reduction in symptom-related disability over the course of the study. The maximum rate of improvement occurred in the first 16 weeks. At week 16, improvement was greatest in the combination arm, next greatest in the SSRI arm and least in the CBT arm, which remained the weakest intervention until the final 52-week endpoint. After 16 weeks, however, the advantage for combined treatment was lost, and, at week 52, the greatest improvement was seen in the SSRI arm.

### Tolerability

A total of 288 AEs were recorded, of which 141 were related to treatment. Three participants (one from each arm) withdrew because of AEs. A total of 11 AEs were judged as ‘severe’ (nine combined and two CBT), with the rest being judged as ‘mild’ or ‘moderate’ severity.

### Safety

Three serious AEs were reported: two (one CBT and one SSRI) were not related to study treatment and involved hospital admissions for termination of pregnancy. The third was a suicide attempt, which was considered to be possibly related to treatment with sertraline monotherapy.

### Economic evaluation

Complete resource use and EQ-5D-3L data were available (at baseline, 16 and 52 weeks) for 23 (46.9%) participants. The mean number of attended sertraline appointments per participant allocated to the sertraline monotherapy group was 4.67, compared with 5.56 for the sertraline plus CBT group. In addition, the mean number of CBT-attended sessions was 6.69 for those allocated to CBT monotherapy (mean session length=101 min/session) and 6.44 for the sertraline plus CBT group (mean session length=106 min/session). As such, the mean annual intervention costs were estimated to be lowest for sertraline monotherapy, followed by CBT monotherapy and sertraline plus CBT. These intervention costs also outweighed other NHS and PSS costs, wherein it is noticeable that, aside from health professional contacts, very few other health care services were used, and no professional carer input was reported (Table 7). Over 52 weeks, the mean EQ-5D-3L scores were estimated to increase for both the sertraline monotherapy and the sertraline plus CBT, but there was a slight fall for the CBT monotherapy group (0.189, 0.205 and −0.016, respectively).

**Table 7:**
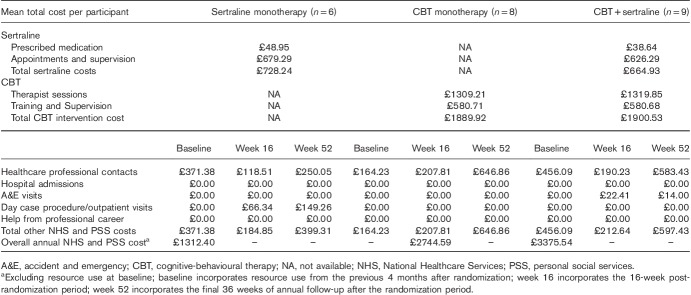
Mean total costs per participant (complete case, estimated annual mean cost per patient)

On the basis of the regression analyses, when compared with sertraline monotherapy, mean costs were £1328.57 (95% CI: £555.39–2101.76) higher for the CBT monotherapy arm and £2175.70 (95% CI: £1385.13–2966.26) higher for the combined arm. The mean QALY scores for sertraline monotherapy were 0.1823 (95% CI: 0.0447–0.3199), greater than that of CBT monotherapy, and 0.1135 (95% CI: −0.0290 to 0.2560), greater than that of the combined arm. As such, sertraline monotherapy was deemed dominant and cost-effective, as it was estimated to be both less costly and more effective than both other options. In addition, according to the cost-effectiveness acceptability curve, at a threshold value of £20 000/QALY, there was estimated to be a 5.3% chance of making the wrong decision by choosing sertraline monotherapy.

## Discussion

Several interventions are available for treating OCD. Few studies, however, have compared the relative effectiveness of these interventions in a single analysis. In addition, given the chronic relapsing nature of OCD, there has been insufficient study of the longer-term treatment outcomes under controlled conditions.

To this end, Skapinakis *et al.* (2016c) recently performed a systematic review and network meta-analysis, comparing all available treatments for adults with OCD, using both direct and indirect data. Fifty-four trials (6652 participants) were included in the network meta-analysis. A shortage of studies comparing active psychological therapy with psychological placebo was noted. The results showed that cognitive-behavioural forms of psychotherapy as well as clomipramine and SSRI (as a class) produced greater improvement in clinical ratings than did pill-placebo therapy. Psychotherapy interventions were reported to be associated with a greater effect than medication, but it was also noted that, in most psychotherapy trials, patients who were taking stable doses of antidepressants were not excluded from the psychotherapy arms. Thus, there was considerable uncertainty about relative effectiveness. The analysis concluded that the combination of behavioural forms of psychotherapy with medications is probably more effective than either monotherapy, at least in the management of severe OCD, and that pragmatic trials with improved research design are needed to establish the differential efficacy between psychotherapies and medications with greater certainty.

The existing uncertainty is of major relevance for health service planning, as NICE guidelines currently recommend either CBT or SSRI monotherapy as first-line approaches, reserving combination treatment for patients with more severe or resistant OCD (National Institute for Health and Care Excellence, 2006). Moreover, in countries such as the UK, CBT is commonly provided for a range of psychiatric disorders including OCD, being delivered in nonmedical psychological therapy service settings, such as the UK Improving Access to Psychological Therapies programme (*https://www.england.nhs.uk/mental-health/adults/iapt/*), wherein medicines management is not always available.

The findings of this feasibility study underline this uncertainty, thus emphasizing the need for a definitive study, and also suggest that running such a study is likely to be feasible and acceptable to patients.

### Feasibility

Recruitment was acceptable across the study centres, with secondary mental healthcare services acting as the principal referral route. Retention to week 8 was also acceptable across all the study arms. Although retention in the combined arm remained good to week 16, a sizeable number of withdrawals occurred after 8 weeks in both the monotherapy groups, suggesting a possible advantage in terms of retention for combined treatment, at least in the acute phase. To maximize the number of evaluable cases, factoring in the unplanned delays related to starting CBT, future studies may aim for a slightly earlier primary endpoint, around 12 weeks. After 16 weeks, fewer patients discontinued, and the majority of patients who had reached week 16 remained in the study until the 52-week endpoint, suggesting that long-term follow-up is feasible for those patients reaching the end of acute-phase treatment.

Study treatment was generally well tolerated and adhered to by the majority of patients. However, four patients randomized to receive sertraline either reduced or stopped it, while another four not randomized to sertraline procured an SSRI prescription from their GP. When questioned, several patients explained that they had found randomization difficult.

Eleven of a total of 288 AEs were considered to be ‘severe’. One patient receiving sertraline attempted suicide, emphasizing the importance of caution in the assessment of suicide risk in OCD patients. No specific association has so far been reported in the scientific literature between the use of SSRI and suicidal acts in adults with OCD. However, an observational cohort study of patients with depression (Coupland *et al.*, 2015) reported increased rates of suicidal behaviour in the first 28 days of starting and stopping antidepressants, highlighting the need for careful monitoring of patients receiving SSRI during these periods.

### Effectiveness

As the number of patients completing 16 weeks fell short of the study power set in the protocol, the findings are subject to type I error, and we cannot be confident that the observed effect is reliable. Therefore, caution is required when interpreting the study outcomes. Notwithstanding, CBT fell considerably short of SSRI in terms of clinical and cost-effectiveness on the observed case analyses, emphasizing the persisting need for a definitive study. On the primary Y-BOCS analysis, at the primary 16-week endpoint, patients receiving CBT were responding less well than those receiving sertraline (Cohen’s *d*=0.27) or sertraline in combination with CBT (Cohen’s *d*=0.39), suggesting that the combined treatment arm may offer the most clinically effective treatment, especially over CBT monotherapy. These findings align with those from two small historic placebo-controlled studies in adults with OCD, one published by Hohagen *et al.* (1998), in which SSRI combined with multimodal behaviour therapy outperformed the psychological therapy given alone on a number of clinical outcomes measures including obsessions and depression, and one published by Cottraux *et al.* (1993), in which fluvoxamine and exposure therapy were synergistic, with an advantage for combined treatment over exposure therapy on rituals at week 8 and on depression at week 24.

If substantiated in a larger trial, our finding of superior effectiveness for sertraline, either in combination with CBT or as a monotherapy, would cast question on the existing evidence-based treatment guidelines (National Institute for Health and Care Excellence, 2006) that tend to recommend CBT or SSRI monotherapy as equivalent first-line treatments. Moreover, the finding that combination treatment may be the most efficacious in this study sample, at least in the short term (≤16 weeks), would suggest that combination treatment should not necessarily be reserved for the most severe and treatment-resistant patients.

Beyond week 16, falling retention across all groups made interpretation exceedingly difficult. However, the advantages of combination therapy were not sustained. Sertraline monotherapy showed the greatest improvement in the Y-BOCS at week 32 and week 52, outperforming both CBT monotherapy with a large effect size (Cohen’s *d*=0.57 and 0.56, respectively) and also outperforming combination treatment (Cohen’s *d*=0.49 and 0.44, respectively).

The failure of combined treatment to show a sustained advantage beyond 16 weeks is, on the face of it, difficult to explain. The gains seen for CBT monotherapy after 16 weeks were slight, but those for SSRI monotherapy were more robust, suggesting that combination with CBT may somehow interfere with the effect of SSRI treatment. Of note, the mean prescribed sertraline dose was rather low overall, considering 200 mg/day is accepted as the optimal dose, which may have reduced efficacy in both the sertraline monotherapy and combined treatment arms. However, this was more noticeable in the combined arm, suggesting patients receiving CBT may have experienced even more difficulties in taking sertraline at optimized doses. This could provide an explanation as to why they failed to improve to the same extent as their monotherapy counterparts. In the combination arm, patients and their clinicians may have biased the focus of treatment towards the adjunctive CBT and held back from taking the maximum sertraline dose. The possibility that a negative interaction exists between receiving CBT and medication should be investigated in more depth, as this would have important treatment implications. Perhaps also some of the gains associated with the combination arm depended upon nonspecific therapist effects that were missed after 16 weeks, when regular contact with the CBT therapist came to an end. Clarification of these factors should be pursued through quantitative and qualitative analysis in the substantive study.

The secondary clinical outcomes largely aligned with the Y-BOCS data, providing a degree of convergent validity to the findings. Specifically, changes in the clinical global severity and improvement scores and the SDS largely mirrored the Y-BOCS data, at least up to week 16, with numerical advantages seen for the combined treatment and SSRI arms over CBT. In the case of the SDS, after 16 weeks, the advantages of combined treatment waned, and, by week 52, sertraline showed the greatest improvement and CBT the least.

Sertraline monotherapy produced the most beneficial effect on depressive symptoms, with the mean baseline MADRS improving by 50% as early as 8 weeks of treatment. In contrast, CBT was associated with no improvement in depressive symptoms: mean MADRS scores at both endpoints were numerically higher than at baseline. This finding runs contrary to a large meta-analysis study of anxiety disorders and OCD, which found that CBT significantly reduced depression in patients with OCD (Hofmann and Smits, 2008). Perhaps by adhering strictly to the exposure and response prevention model, patients receiving CBT experienced greater levels of distress that manifested as increased MADRS scores. This may also explain the relatively reduced ‘antidepressant’ effect when sertraline was combined with CBT, compared with sertraline monotherapy.

### Health economic analysis

Despite the falling numbers and relatively low response rates (49%), a preliminary health economic analysis was possible. Sertraline monotherapy was estimated to be associated with both a higher mean QALY gain and lower mean costs, when compared with CBT monotherapy and sertraline+CBT. These cost-effectiveness results had associated uncertainty and should again be treated with caution due to the relatively small sample size. In addition, they were based on a complete-case approach, and such individuals may not be representative of all participants. However, we have conducted sensitivity analyses (results available from authors), including multiple imputations (Faria *et al.*, 2014), to account for missing cost and outcome data, and, in each case, sertraline monotherapy was estimated to be cost-effective, compared with the other two treatment options.

We are aware of two previous publications (National Collaborating Centre for Mental Health, 2006; Skapinakis *et al.*, 2016b) looking at the cost-effectiveness of CBT in OCD patients compared with SSRIs (at a class level including sertraline) and CBT and SSRIs combined. One (Skapinakis *et al.*, 2016b) estimated that that SSRIs were more cost-effective, whereas the other (National Collaborating Centre for Mental Health, 2006) estimated that the combined option was the most cost-effective.

However, both were model-based studies based on a number of assumptions, some of which, for example, therapy frequency and session length, were not always explicitly stated. Accordingly, explaining the reasons for the differing results is not straight forward.

In this study, it was notable that total other NHS and PSS costs were outweighed by the intervention costs in all three groups, and that, within this cost category, health professional contacts were by far the main cost driver. Accordingly, resource items such as professional carer time and accident and emergency visits seem unlikely to have a notable influence in any future study, and there is an argument that these need not be measured, as it would reduce patient burden. In turn, this may improve response rates, which were lower than expected, possibly due to the requirement for in-person follow-up. In addition, our study followed-up patients under controlled conditions for 12 months only.

Considering the high relapse rates seen in patients receiving treatment for OCD over 5 years (Eisen *et al.*, 2013), a longer-term follow-up period may be needed to thoroughly review the cost-effectiveness of treatment for this chronic debilitating disorder.

### Advantages and limitations

The advantages of this study are the randomized design, blinded raters, the exclusion of concomitant antidepressants, the ITT and observed case analysis, and the use of accepted efficacy scales and pharmacoeconomic assessments. The application of robust methodology is important in a field where less careful methodology is unfortunately common. Limitations include the low numbers of participants, high dropout rates, low dosage of sertraline and the use of some assumptions to calculate the duration of healthcare contact in the different groups. Observed case analysis is sensitive to the effect of treatment in those participants who are known to be taking it. Although this form of analysis is useful for feasibility studies, it is likely to overestimate the true effect size, and therefore cannot be considered definitive, as the analysis is unable to take account of the effect of treatment on those participants who failed to undergo routine evaluation. In this study, the findings of superiority for SSRI on the Y-BOCS were not substantially changed by the ITT analysis. At 16 weeks, the effect size in favour of sertraline, or combined therapy over CBT, is larger than for the observed cases, but, by 32 weeks, the pattern is very similar to the observed cases. Nevertheless, caution is required in interpreting our observed case results, given the lack of study power, and argues in favour of performing a large, definitive study.

### Conclusion

Recruitment was feasible across the three study arms in three centres, and the study procedures were acceptable to the majority of patients. Retention was acceptable across the three study arms up to week 8, and in the combined arm up to week 16. To maximize the number of evaluable cases, future studies may aim for a primary endpoint around 12 weeks.

Longer-term participant retention was adequate, with the majority of those who had reached week 16 remaining in the study until the 52-week endpoint, suggesting that long-term follow-up is feasible for those patients reaching the end of acute-phase treatment.

At weeks 8 and 16, sertraline-treated patients responded better than those receiving CBT in the observed case analyses. The combined arm appeared to offer the most clinically effective treatment (especially over CBT) in the acute treatment phase. Beyond week 16, falling retention made interpretation difficult, but several analyses including a preliminary health economic analysis suggested that there were ongoing advantages for receiving sertraline relative to CBT.

### Implications

Were these findings to be substantiated in a more definitive study, that is, if sertraline monotherapy were to be associated with greater sustained efficacy and lower costs than usual care with CBT, there would be the potential for changes to existing treatment guidelines with resulting large cost savings to the NHS. Further research would, therefore, be of value: our study confirms that a definitive study can and should be conducted.
